# Impact of Humidity on Quartz-Enhanced Photoacoustic Spectroscopy Based CO Detection Using a Near-IR Telecommunication Diode Laser

**DOI:** 10.3390/s16020162

**Published:** 2016-01-27

**Authors:** Xukun Yin, Lei Dong, Huadan Zheng, Xiaoli Liu, Hongpeng Wu, Yanfang Yang, Weiguang Ma, Lei Zhang, Wangbao Yin, Liantuan Xiao, Suotang Jia

**Affiliations:** 1 State Key Laboratory of Quantum Optics and Quantum Optics Devices, Institute of Laser Spectroscopy, Shanxi University, Taiyuan 030006, China; yinxkvip@163.com (X.Y.); zhenghuadan@126.com (H.Z.); liuxiaoli@163.com (X.L.); beijing2008whp@163.com (H.W.); 15735176489@163.com (Y.Y.); mwg@sxu.edu.cn (W.M.); k1226@sxu.edu.cn (L.Z.); ywb65@sxu.edu.cn (W.Y.); xlt@sxu.edu.cn (L.X.); tjia@sxu.edu.cn (S.J.); 2 Collaborative Innovation Center of Extreme Optics, Shanxi University, Taiyuan 030006, China; 3 State Key Laboratory on Integrated Optoelectronics, Jilin University, Changchun 130012, China

**Keywords:** quartz enhanced photoacoustic spectroscopy, carbon monoxide, vibrational-to-translational (V–T) relaxation, near-IR telecommunication diode laser

## Abstract

A near-IR CO trace gas sensor based on quartz-enhanced photoacoustic spectroscopy (QEPAS) is evaluated using humidified nitrogen samples. Relaxation processes in the CO-N_2_-H_2_O system are investigated. A simple kinetic model is used to predict the sensor performance at different gas pressures. The results show that CO has a ~3 and ~5 times slower relaxation time constant than CH_4_ and HCN, respectively, under dry conditions. However, with the presence of water, its relaxation time constant can be improved by three orders of magnitude. The experimentally determined normalized detection sensitivity for CO in humid gas is $1.556\times 10^{-8}\;W\cdot {\text{cm}}^{-1}/{\text{Hz}}^{1/2}$.

## 1. Introduction

Photoacoustic spectroscopy (PAS) has been one of the most widely used spectroscopic techniques for trace gas detection in the past decades because of its advantages of high sensitivity, high selectivity and compact detection module. The principle of PAS is to detect the sound waves which are generated in the media upon absorption of modulated optical radiation. The optical radiation initially provides the vibrational excitation of molecules and then the excited states lose their energy by vibration-translation relaxation (V-T relaxation), resulting in heating and thermal expansion of the local gas. Periodic pressure waves, *i.e.*, acoustic waves, are produced and subsequently detected by highly sensitive microphones. Quartz-enhanced photoacoustic spectroscopy (QEPAS), an alternative approach to PAS utilizing a quartz tuning fork (QTF) as a sharply resonant acoustic transducer instead of a microphone [1,2,3], has been widely applied to environmental monitoring, industrial process control, combustion processes analysis, and medical diagnosis [4,5,6,7,8,9,10,11,12,13,14]. Compared with the microphone-based PAS which employs the modulation frequency of 1–4 kHz determined by the acoustic resonances modes of the photoacoustic cells, the QEPAS employs the higher modulation frequency of ~32 kHz, corresponding to the oscillation frequency of the commercial QTFs. Such a high modulation frequency makes the QEPAS technique immune to 1/*f* and environmental acoustic noise. However, a stringent condition for PAS which has to be taken into account is that the molecular relaxation time τ should be shorter than the modulation period, *i.e.*, τ≪1/f [15], because otherwise it can cause signal amplitude reduction or phase shifts of the photoacoustic signal when using QEPAS to detect the molecules with a slow V-T relaxation [15,16]. The V-T relaxation processes of CH_4_, HCN and CO_2_ have been widely investigated using the QEPAS. However, the V-T relaxation process of carbon monoxide (CO) and the influence of H_2_O on the CO V-T energy transfer have not been investigated by means of the QEPAS so far. Previous studies on CO QEPAS sensors [10] were focused on the improvement of CO detection sensitivities. Therefore, the motivation of this paper is to investigate the V-T relaxation process of CO and the influence of H_2_O on the CO V-T relaxation energy transfer in detail using the QEPAS sensor system, which is very important for the QEPAS-based CO sensing. It is well known that CO has a slow V-T relaxation. Carbon monoxide is the product of incomplete combustion of organic matter due to insufficient oxygen (O_2_) supply. As a colorless and odorless poisonous gas, ~100 ppm level CO can cause danger to the human central nervous system and heart [17]. Therefore, the study of the CO V-T relaxation process is important for the CO QEPAS sensor design and optimization.

Near-IR telecommunication diode lasers have been widely used in spectroscopic technology due to their narrow line-width, fast tuning rate, stable emitting wavelength, and long life [18,19,20]. Moreover, compared with the mid-infrared quantum cascade lasers (QCLs) or far-infrared Terahertz (THz) lasers [21,22,23], near-IR telecommunication lasers are more cost-effective. Although the absorption of the overtone band of molecules in the near-IR region is weaker than that of the fundamental band in the mid-IR region, the sensitivity loss can be compensated by boosting the laser power [24,25,26], due to the fact that PAS sensitivity is proportional to the optical excitation power. In this work, a near-IR QEPAS sensor was developed to investigate the CO V-T relaxation in the dry or wet nitrogen (N_2_) gas mixtures respectively, by use of a 1.57 µm near-IR distribute feedback (DFB) laser source. The QEPAS sensor performance under different humidities and pressures were evaluated in detail by experimental investigation and theoretical simulation.

## 2. Experimental Setup

**Figure 1:**
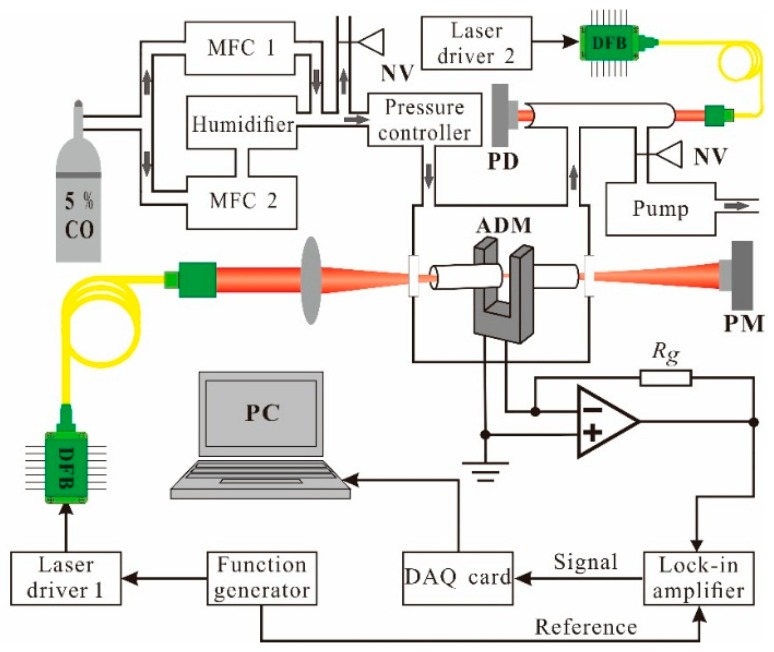
Schematic diagram of the experimental setup. MFC: mass flow controller; PM: power meter; PD: photo detector; NV: needle valve; ADM: acoustic detection module; PC: personal computer.

A schematic of the experimental setup is shown in Figure 1. A fiber-coupled distributed feedback (DFB) diode laser with an emitting wavelength centered at 1566.31 nm (Model PN: DFB-914010C1424-42, Sichuan Tengguang Electronics and Technology Co., Chengdu, China), was used to excite photoacoustic signals.

**Figure 2:**
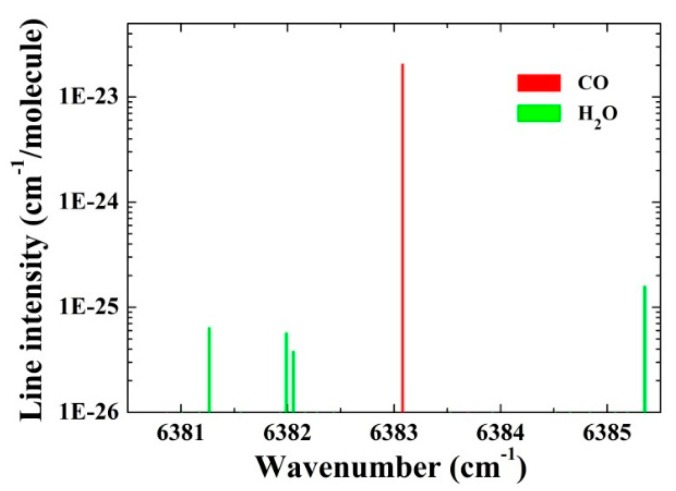
Absorption lines of CO and H_2_O between 6380.5 cm^−1^ and 6385.5 cm^−1^.

The laser current and temperature were controlled by a control electronics unit (CEU) [27]. The current of the DFB laser was modulated at the half resonance frequency of the QTF by the CEU. The laser wavelength can be tuned from 6377.41 cm^−1^ to 6388.35 cm^−1^ by changing the laser temperature from 36.45 °C to 8.45 °C with a constant current of 100 mA. According to the HITRAN database [28], the molecular absorption lines for CO and H_2_O within the wavelength range of 6380.5 cm^−1^ to 6385.5 cm^−1^ are shown in Figure 2.

The CO absorption line located at 6383.1 cm^−1^, is two orders of magnitude higher than that of neighbor H_2_O lines, indicating that the target absorption line is interference-free from the H_2_O absorption lines and it can be used to investigate the influence of H_2_O on the CO V-T relaxation using the QEPAS technique. With a constant laser temperature of 21.95 °C, the laser current was scanned from 88 mA to 108 mA, corresponding to 6383.3 cm^−1^ to 6382.9 cm^−1^, in order to cover the selected CO absorption line. The output laser beam was focused to pass through the QEPAS spectrophone with a beam waist radius of 50 µm by a fiber focuser (Model PN: 163426-0, OZ Optics, Ottawa, ON, Canada). A commercially available QTF with a resonance frequency (*f*) of 32.755 kHz and quality factor (*Q*-factor) of 13,534, measured in the atmosphere, was used as the photoacoustic transducer. The QEPAS spectrophone was constructed in an on-beam spectrophone configuration [29], including two identical metallic tubes with the length of 4.0 mm, the inner diameter of 0.8 mm and the outer diameter of 1.24 mm. The resonance frequency and *Q*-factor of the on-beam QEPAS spectrophone were measured to be *f* = 32755.4 and *Q* = 3432. The optimum on-beam QEPAS spectrophone can offer a signal-to-noise ratio (SNR) enhancement factor of ~30. The output signal from the QTF was processed by a low noise trans-impedance amplifier (TPA) with a feedback resistor of *R_g_* = 10 MΩ, and then led to a lock-in amplifier (Model: SR830, Stanford Research Systems, Inc., Sunnyvale, CA, USA) to demodulate the signal in 2*f* mode. The lock-in amplifier was set to a filter slope of 12 dB/oct and a time constant of 300 ms, which corresponds to a detection bandwidth ∆*f* = 0.833 Hz. A computer with a NI DAQ card (Model: NI PCI-6251, National Instrument, Austin, TX, USA) was used to acquire and process all the data of this QEPAS sensor.

The gas sample with 5% CO in N_2_ was divided into two gas lines, which were directed to two mass flow controllers (MFCs) (Model: CS200(A), D07-19B, Beijing Seven Star Electronics Co., Beijing, China), respectively. One gas line controlled by MFC #2 was humidified by a humidifier (Model: MH-110-24F-4, Perma Pure., Lakewood, NJ, USA). The other gas line controlled by MFC #1 merged with the humidified gas line to generate gas mixtures with different humidities. The pressure in the system was controlled by a pressure controller (Model: 649B13TS1M22M, MKS Instruments Inc., Andover, MA, USA) and a vacuum pump (Model: D16C, Oerlikon Leybold Vacuum Inc., Cologne, Germany). A 50-cm long gas absorption cell was installed after the acoustic detection module (ADM) to monitor the H_2_O concentration by means of direct absorption spectroscopy implemented by a DFB diode laser (Model: DFB-136813C1424, Chengdu Huawin Laser Co., Sichuan, Chengdu, China,) emitting at 7306.75 cm^−1^ and a photo detector (Model: GT-51084-12N12, GlobTek, Inc., Northvale, NJ, USA). The gas flow rate of the system was set at 70 standard-state cubic centimeters per minute (sccm).

## 3. Results and Discussion

### 3.1. Optimization of Modulation Amplitude and Pressure in Dry and Wet Gas Samples

**Figure 3:**
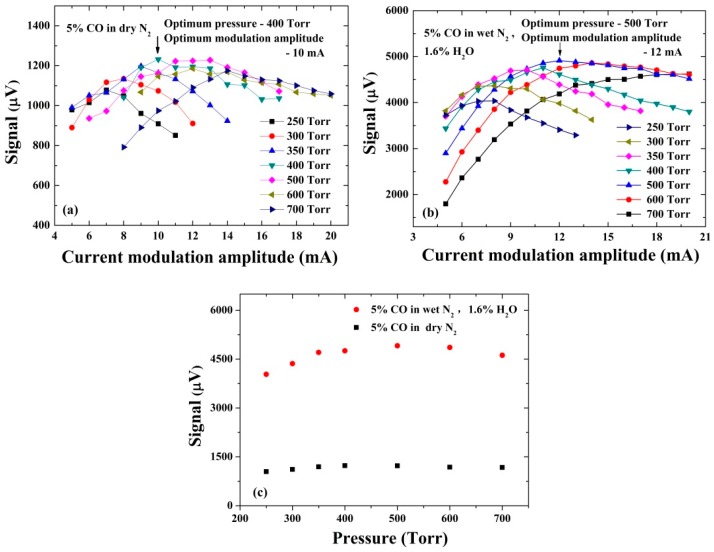
(**a**,**b**) 2*f* QEPAS signal amplitudes obtained at different pressures and laser modulation amplitudes in dry and wet 5% CO/N_2_ mixtures, respectively; (**c**) 2*f* QEPAS signal amplitudes for optimum modulation amplitude at different gas pressures.

As the QEPAS is a 2*f* wavelength modulation based technique, the sensor performance depends on the modulation amplitude of the laser source and the gas pressure. With a low optical excitation power, the QEPAS signal can be expressed as [25]:

(1) $$S\left(P\right)=K\cdot P\cdot C\cdot Q\left(P\right)\cdot \alpha_{0}\left(P\right)\cdot \varepsilon \left(P\right)$$

where *K*, *P* and *C* are the sensor constant, the incident optical power and the target gas concentration, respectively. These parameters are independent of the pressure (*P*). The Q(P), $\;\alpha_{0}\left(P\right)$ and ε(P) are the quality factor (*Q*-factor) of QTF, the peak of 2*f* absorption spectrum , and the conversion efficiency ε of the absorbed optical power into the sound, respectively. Q, $\alpha_{0}$ and ε are pressure dependent. Moreover, the laser wavelength modulation must match the pressure-dependent absorption linewidth. The highest $\alpha_{0}$ is achieved when the modulation amplitude is close to the absorption linewidth [26]. Therefore, the sensor performance was evaluated by changing the gas pressure of the system and the wavelength modulation amplitude of the laser. Figure 3a,b show that the modulation amplitude of the laser current was optimized at different pressures with dry and humid gas samples, respectively. The 2*f* QEPAS signal amplitudes for the dry 5% CO/N_2_ mixture are depicted in Figure 3a, while the 2*f* QEPAS signal amplitudes for the 5% CO/N_2_ mixture with 1.6% water are depicted in Figure 3b. The water concentration was measured via direct absorption spectroscopy, as shown in Figure 1. The *Q*-factor and the resonance frequency of the QTF were actively measured by the CEU.

Figure 3c shows the dry and wet QEPAS signal amplitudes for the optimized modulation amplitude at different gas pressures. As shown in Figure 3a,c, the maximum QEPAS signal amplitude in the dry 5% CO/N_2_ mixture, is obtained at the pressure of 400 Torr with a modulation amplitude of 10 mA. The variation of QEPAS signals at the pressures range 250–700 Torr is <10%. However, a significant increase of the signal amplitude is observed when water is added to the gas mixture. The maximum QEPAS signal amplitude in the wet 5% CO/N_2_ mixture is obtained at 500 Torr with a modulation amplitude of 12 mA, which is ~4 times higher than that obtained in the dry condition. The significant increase of the 2*f* QEPAS signal amplitudes indicates that the H_2_O molecules behave as a signal promoter to enhance the QEPAS signal effectively [10,30].

### 3.2. Investigation of the V-T Relaxation: CO in Dry N_2_

The CO has a slow V-T energy relaxation process and as a result, the relaxation rate cannot effectively follow the modulation frequency of 32 kHz in the QEPAS [10,30]. The discrepancy between the V-T relaxation rate and the modulation frequency usually results in the signal amplitude reduction and phase shift. An alternative approach to improve the relaxation rate is to increase the translation motion of the molecules by improving the gas pressure. However, according to Equation (1), the quality factor Q(P), the peak absorption value $\alpha_{0}\left(P\right)$, and the conversion efficiency ε(P) are pressure dependent. In order to further investigate the V-T process of CO, it is necessary to study Q(P), $\alpha_{0}\left(P\right)$ and ε(P) first. For simplicity, the sensor constant K was assumed to be 1 in the following sections.

#### 3.2.1. *Q*-Factor Measurement

**Figure 4:**
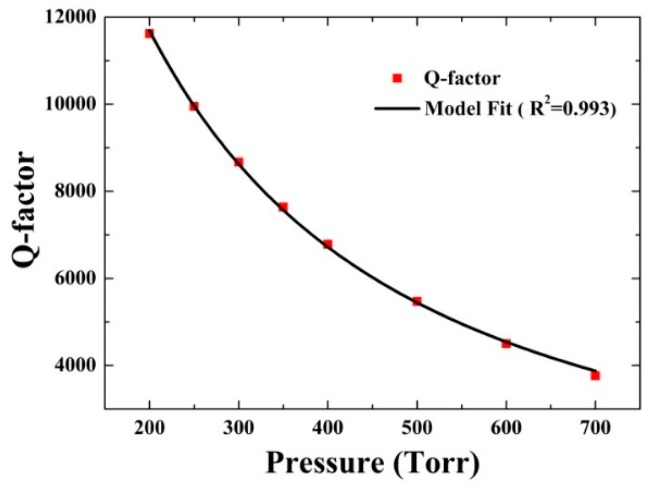
*Q*-factor of the QTF as a function of the gas pressures.

A QTF oscillator can be equivalent to a RLC series circuit according to the reference [2,31], and the QTF parameters can correspond to equivalent electrical parameters: mass (*m*) to inductance (*L*), rigidity (*k*) to inverse capacity (1/*C*) and damping to resistance (*R*). The Q(P) of the QTF can be described by the expression [2,31]:

(2) $$Q\left(p\right)=\frac{Q_{vac}}{1+Q_{vac}aP^{b}}$$

where $Q_{vac}$ is the *Q*-factor in vacuum, *P* is the gas pressure expressed in Torr, *a* and *b* are the parameters specific to a particular kind of QTF, respectively. The $Q_{vac}$ of the used QTF was measured to be 26,029, before removal of the metallic housing of the QTF. The variation of its *Q*-factor as a function of the gas pressures was measured and plotted in Figure 4.

The *Q*-factor decreases monotonously with the increase of the gas pressure. Equation (2) was used to fit the data. The obtained fitting parameters are $a=7.004\times 10^{-8}\pm 5.749\times 10^{-9}$ and b=1.229±0.014. The *R* square of >0.99 indicated a good relevancy.

#### 3.2.2. Simulation of the Peak $\alpha_{0}\left(P\right)$ of 2*f* Absorption Spectrum 

**Figure 5:**
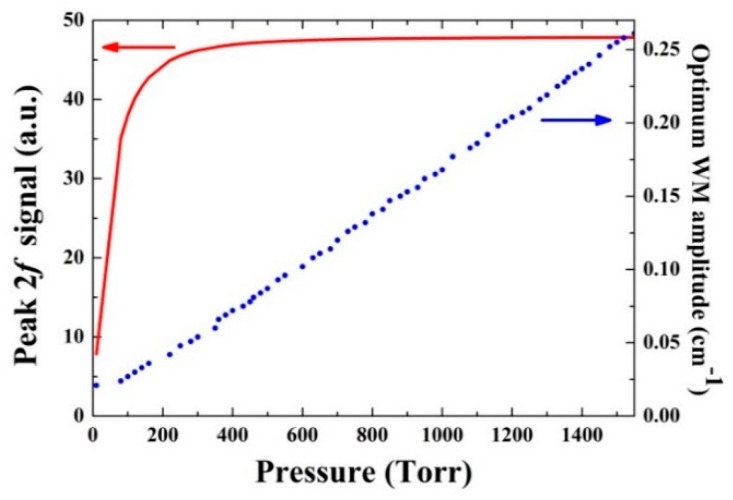
Optimum wavelength modulation amplitude A(P) (circles) and the corresponding CO 2*f* absorption peak $\alpha_{0}\left(P\right)$ (line) as a function of the gas pressures.

According to Section 3.1, the pressure *P* and the laser wavelength modulation amplitude *A* have a crucial impact on the QEPAS signal amplitude. The simulation of the CO 2*f* wavelength modulation at different pressures can be used to obtain$\;\alpha_{0}\left(P\right)$. The CO line intensity as well as the broadening coefficients, can be found from the HITRAN database [28]. The self-broadening coefficient of CO was neglected in the simulation, because of its low concentration. The optimum modulation amplitude A(P) and the corresponding 2*f* absorption peak $\alpha_{0}\left(P\right)$ in the gas pressure range 0–1550 Torr are presented in Figure 5. The numerical calculation indicated that modulation amplitude corresponding to the highest 2*f* signal is approximately 2.2 times the half width at half maximum (HWHM) of the Lorentzian-shaped absorption line [32,33].

#### 3.2.3. Calculation of the Conversion Efficiency ε(P)

**Figure 6:**
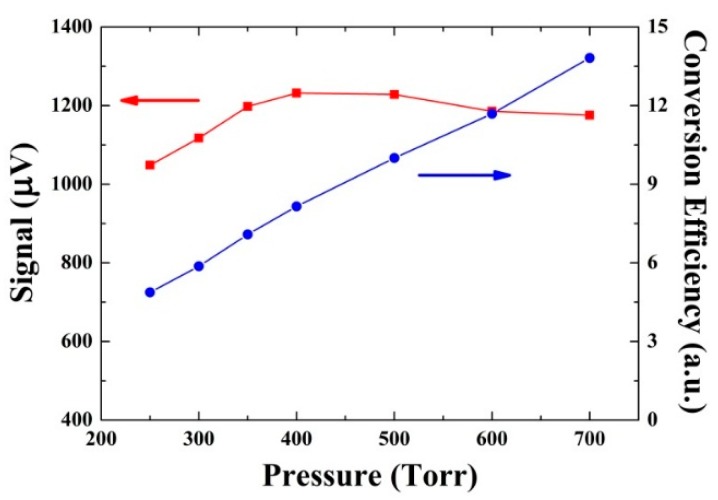
Measured CO QEPAS signals (squares) and corresponding conversion efficiency *ε* (*P*) (circles) as a function of the gas pressure.

With the obtained Q(P), $\alpha_{0}\left(P\right)$ and the experimentally measured signals S(P), the ε(P) can be calculated according to Equation (1). The measured S(P) with optimum modulation amplitude and calculated ε(P) at different pressures are plotted in Figure 6. It can be observed that the conversion efficiency increases monotonously with the pressures increasing. This can be attributed to the more drastic collision of molecules at the higher pressure, which means a faster V-T energy transfer. However the maximum signal was obtained at 400 Torr. This is due to the fact that the decrease of the *Q*-factor starts to dominate the 2*f* signal amplitude beyond 400 Torr although the conversion efficiency between 250 and 700 Torr continues increasing.

According to reference [32], the conversion efficiency ε(P) can be expressed as:

(3) $$\varepsilon \left(P\right)=\frac{1}{\sqrt{1+{\left[\frac{2\pi \cdot f\cdot P_{0}\cdot \tau_{0}^{N}}{P}\right]}^{2}}}$$

**Figure 7:**
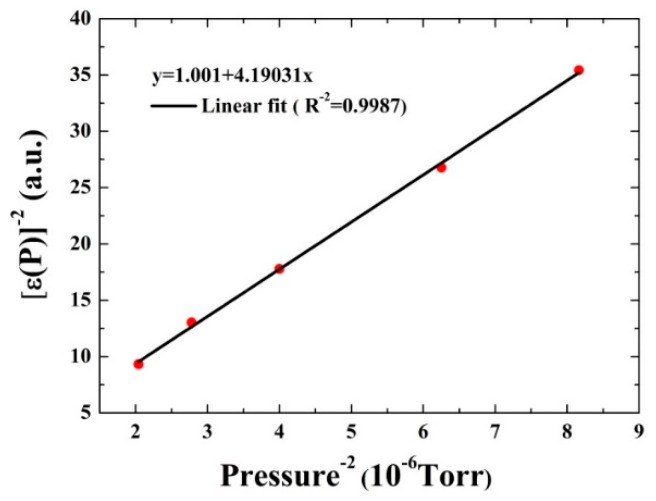
Experimental results (circles) and linear fit for determining the V-T relaxation rate of CO in dry N_2_.

In order to achieve a linear fit, Equation (3) is rewritten as:

(4) $${\left[\frac{1}{\varepsilon \left(P\right)}\right]}^{2}=1+{\left(2\pi \cdot f\cdot P_{0}\cdot \tau_{0}^{N}\right)}^{2}\cdot \frac{1}{P^{2}}$$

where *f* and $P_{0}\tau_{0}^{N}$ represent the modulation frequency and the V-T relaxation time constant, respectively. In this way, the relaxation time constant can be determined by the slope$\;{\left(2\pi \cdot f\cdot P_{0}\cdot \tau_{0}^{N}\right)}^{2}$. The variation of conversion efficiency ε(P) as a function of $\;1/P^{2}$ is shown in Figure 7. A linear fitting was carried out and an *R* square value of 0.998 was obtained. The relaxation time constant $P_{0}\tau_{0}^{N}$ of CO calculated from the fitting slope is 9.95±0.07 ms Torr in the dry N_2_, which is ~3 and ~5 times slower than the relaxation time constant of CH_4_ of 2.9±0.2 ms Torr and HCN of 2.2±0.4 ms Torr in dry N_2_, respectively [16,32]. Moreover the V-T relaxation time $\tau_{0}^{N}$ of CO in dry N_2_ was calculated to be 25 µs by dividing the $P_{0}\tau_{0}^{N}$ by the gas pressure $\;P_{0}$, which is comparable with the QEPAS modulation period of ~30 µs. This implies that a dry CO/N_2_ mixture is not suitable for the QEPAS technique to determine the CO concentration.

**Figure 8:**
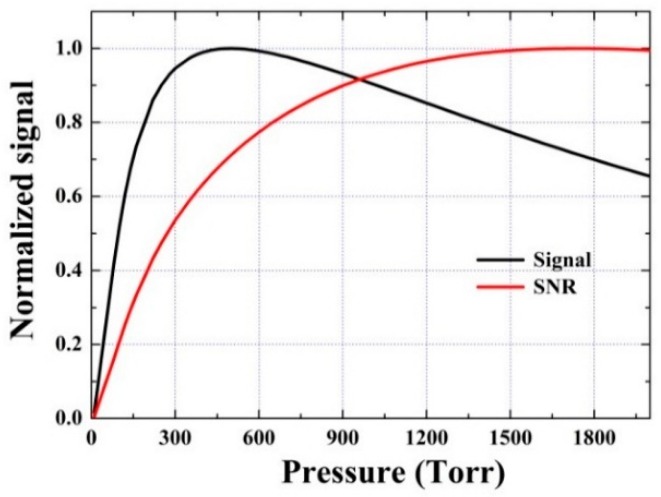
QEPAS sensor performances in terms of signal amplitudes and SNR predicted by the developed relaxation model for detecting CO in dry N_2_.

With the obtained Q(P), $\alpha_{0}\left(P\right)$ and ε(P), Equation (1) can be used to predict the signal S(P) and signal-to-noise ratio ($SNR\sim S\left(P\right)/\sqrt{Q\left(P\right)}\;$) at a given concentration of CO/N_2_ mixture [29]. The predicted signal amplitudes and the corresponding SNR in the pressure range 0–2000 Torr are presented in Figure 8. The predicted strongest signal is obtained at 500 Torr, which is close to the result of 400 Torr obtained by the developed QEPAS sensor. The highest SNR will be obtained at 1750 Torr according to the developed kinetic model.

### 3.3. Investigation of the V-T Process: CO in Wet N_2_

In order to investigate the V-T relaxation process of CO in wet N_2_, a simplified theoretical model of CO V-T relaxation was developed, assuming that only one-stage collisions between molecules occurs [16]. The obtained QEPAS signal in the wet N_2_ consists of two parts: the collision of CO and N_2_ molecules, and CO and H_2_O molecules, which are described by the signals$\;S_{1}$ and$\;S_{2}$, respectively. The signal *S_2_* describing the CO/H_2_O collisions depends on H_2_O partial pressure $P_{H}$. The collisions of two CO molecules are neglected because of the low concentration of CO. The QEPAS signal with varying H_2_O concentrations, expressed as$\;S\left(P_{H}\right)=S_{1}+S_{2}\;$, was measured at 500 Torr. Assuming that the part of the initial vibrational excitation energy released via CO/N_2_ collisions (*S_1_*) remains constant, according to Equation (3), the signal $S\left(P_{H}\right)$ can be described as:

(5) $$S\left(P_{H}\right)=S_{1}+S_{2}=S_{1}\left[1+\frac{\eta -1}{\sqrt{1+{\left(\frac{2\pi \cdot f\cdot P_{0}\cdot \tau_{0}^{H}}{P_{H}}\right)}^{2}}}\right]$$

where η=S(∞)/S(0), S(∞) and S(0) are equal to the maximum signals obtained with the saturated water and in dry N_2_ at 500 Torr gas pressure, respectively. Here we assume that the water in the CO/N_2_ mixture is saturated, and a η value of 4 was obtained. The relaxation time constant $P_{0}\tau_{0}^{H}$ describes the V-T relaxation due to CO/H_2_O collisions. If$\;\left(\frac{2\pi \cdot f\cdot P_{0}\cdot \tau_{0}^{H}}{P_{H}}\right)\gg 1$, Equation (5) can be changed to be a linear equation:

(6) $$S\left(P_{H}\right)\approx S_{1}\left[1+\frac{\eta -1}{2\pi \cdot f\cdot P_{0}\cdot \tau_{0}^{H}}P_{H}\right]$$

**Figure 9:**
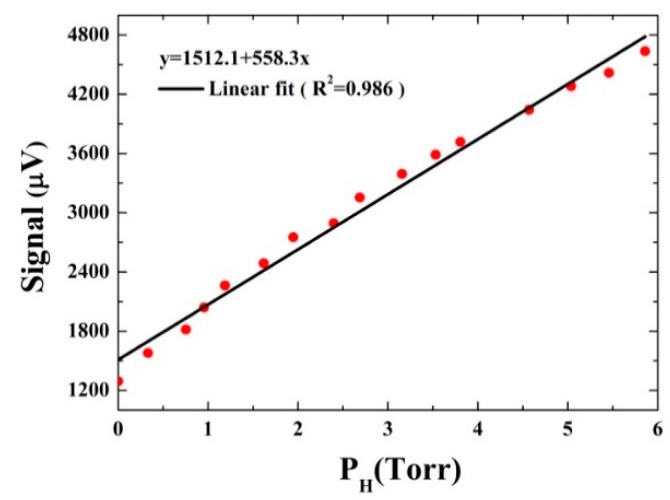
QEPAS signal (circles) as a function of H_2_O concentration for detecting CO in wet N_2_.

The QEPAS signal obtained at different H_2_O partial pressure and the corresponding linear fitting results are shown in Figure 9. A relaxation time constant $P_{0}\tau_{0}^{H}$ of 39.49±1.95 μs Torr for CO in wet N_2_ was calculated from the slope of the fitting curves. And the corresponding V-T relaxation time $\tau_{0}^{H}$ of 0.078 µs was obtained. Assuming that the influence of water concentrations on Q(P) and $\alpha_{0}\left(P\right)$ can be neglected, the conversion efficiency and corresponding QEPAS signals can also be determined at given water concentration and gas pressure, according to Equations (1) and (5).

**Figure 10:**
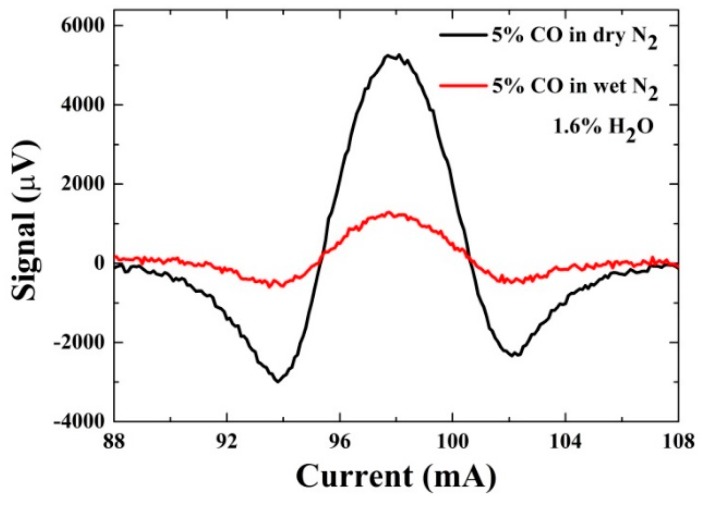
QEPAS signals obtained in the dry N_2_ and wet N_2_ as a function a laser current.

The profile of QEPAS signals obtained in dry and wet 5% CO/N_2_ mixture are shown in Figure 10. The laser current was scanned from 88 to 108 mA to cover the CO absorption line located at 6383.1 cm^−1^. For the detection of CO in dry N_2_, a detection SNR of 22.3 was obtained with 300 ms averaging time at 400 Torr, which corresponds to a normalized noise equivalent absorption coefficient (NNEA) of $5.92\times 10^{-8}\;W\cdot {\text{cm}}^{-1}/{\text{Hz}}^{1/2}$. However the detection SNR of 84.2 was obtained in the wet N_2_ at 500 Torr, which is nearly four times higher than that obtained in the dry N_2_ and corresponds to a NNEA of $1.556\times 10^{-8}\;W\cdot {\text{cm}}^{-1}/{\text{Hz}}^{1/2}$. This is due to the fact that the V-T relaxation time of the CO in wet N_2_ at 500 Torr was calculated as 0.078 µs which was shorter than V-T relaxation time of 24.8 µs obtained in dry N_2_ at 400 Torr.

## 4. Conclusions

A near-IR QEPAS-based trace gas sensor was developed to investigate the molecular V-T relaxation of CO by photoacoustic detection. The performance of the QEPAS sensor was evaluated by experimental investigation and theoretical simulation under the different humidity and pressure conditions. A normalized detection sensitivity of $1.556\times 10^{-8}\;W\cdot {\text{cm}}^{-1}/{\text{Hz}}^{1/2}$ for CO in humid gas was achieved when 1.6% water was added into the gas mixture. The relaxation time constant $P_{0}\tau_{0}^{N}$ in the dry N_2_ and $P_{0}\tau_{0}^{H}$ in wet *N*_2_ are 9.95±0.07 ms Torr and 39.49±1.95 μs Torr, respectively. The relaxation time constant was improved by as much as a factor of ~230 with the presence of 1.6% water. Using Equation (3), the QEPAS signal reached 90% of its instantaneous-relaxation value when the partial H_2_O pressure was 14 Torr, which corresponds to 60% relative humidity at +24 °C. Limited by the performance of our humidifier, our wet gas mixture (1.6% water content, 35% relative humidity at +24 °C) did not reach the CO instantaneous-relaxation value. The addition of more water can further improve the CO signal amplitude. Due to the crucial role of water in the V-T energy transfer, the water concentration must be controlled or monitored on-line to calibrate the signal amplitude, so that the concentration of the analyzed CO mixture can be precisely determined.

## References

[1] Kosterev A.A., Bakhirkin Y.A., Curl R.F., Tittel F.K. (2002). Quartz-enhanced photoacoustic spectroscopy. Opt. Lett..

[2] Patimisco P., Scamarcio G., Tittel F.K., Spagnolo V. (2014). Quartz-enhanced photoacoustic spectroscopy: A review. Sensors.

[3] Dong L., Wu H.P., Zheng H.D., Liu Y.Y., Liu X.L., Jiang W.Z., Zhang L., Ma W.G., Ren W., Yin W.B. (2014). Double acoustic micro-resonator quartz enhanced photoacoustic spectroscopy. Opt. Lett..

[4] Sampaolo A., Patimisco P., Dong L., Geras A., Scamarcio G., Starecki T., Tittel F.K., Spagnolo V. (2015). Quartz-enhanced photoacoustic spectroscopy exploiting tuning fork overtone modes. Appl. Phys. Lett..

[5] Zheng H.D., Dong L., Yin X.K., Liu X.L., Wu H.P., Zhang L., Ma W.G., Yin W.B., Jia S.T. (2015). ppb-level QEPAS NO_2_ sensor by use of electrical modulation cancellation method with a high power blue LED. Sens. Actuators B Chem..

[6] Wu H.P., Dong L., Ren W., Yin W.B., Ma W.G., Zhang L., Jia S.T., Tittel F.K. (2015). Position effects of acoustic micro-resonator in quartz enhanced photoacoustic spectroscopy. Sens. Actuators B Chem..

[7] Jahjah M., Jiang W., Sanchez N.P., Ren W., Patimisco P., Spagnolo V., Herndon S.C., Griffin R.J., Tittel F.K. (2014). Atmospheric CH_4_ and N_2_O measurements near Greater Houston area landfills using a QCL-based QEPAS sensor system during DISCOVER-AQ 2013. Opt. Lett..

[8] Liu K., Zhao W.X., Wang L., Tan T., Wang G.S., Zhang W.J., Gao X.M., Chen W.D. (2015). Quartz-enhanced photoacoustic spectroscopy of HCN from 6433 to 6613 cm^−^^1^. Opt. Commun..

[9] Yi H.M., Maamary R., Gao X.M., Sigrist M.W., Fertein E., Chen W.D. (2015). Short-lived species detection of nitrous acid by external-cavity quantum cascade laser based quartz-enhanced photoacoustic absorption spectroscopy. Appl. Phys. Lett..

[10] Ma Y.F., Lewicki R., Razeghi M., Tittel F.K. (2013). QEPAS based ppb-level detection of CO and N_2_O using a high power CW DFB-QCL. Opt. Express.

[11] Cao Y.C., Sanchez N.P., Jiang W.Z., Griffin R.J., Xie F., Hughes L.C., Zah C., Tittel F.K. (2015). Simultaneous atmospheric nitrous oxide, methane and water vapor detection with a single continuous wave quantum cascade laser. Opt. Express.

[12] Ren W., Jiang W.Z., Sanchez N.P., Patimisco P., Spagnolo V., Zah C.-E., Xie F., Hughes L.C., Griffin R.J., Tittel F.K. (2014). Hydrogen peroxide detection with quartz-enhanced photoacoustic spectroscopy using a distributed-feedback quantum cascade laser. Appl. Phys. Lett..

[13] Pohlkotter A., Kohring M., Willer U., Schade W. (2010). Detection of molecular oxygen at low concentrations using quartz enhanced photoacoustic spectroscopy. Sensors.

[14] Ba T.N., Triki M., Desbrosses G., Vicet A. (2015). Quartz-enhanced photoacoustic spectroscopy sensor for ethylene detection with a 3.32 μm distributed feedback laser diode. Rev. Sci. Instrum..

[15] Wysocki G., Kosterev A.A., Tittel F.K. (2006). Influence of molecular relaxation dynamics on quartz-enhanced photoacoustic detection of CO_2_ at λ = 2 μm. Appl. Phys. B.

[16] Kosterev A.A., Mosely T.S., Tittel F.K. (2006). Impact of humidity on quartz-enhanced photoacoustic spectroscopy based detection of HCN. Appl. Phys. B.

[17] Prockop L.D., Chichkova R.I. (2007). Carbon monoxide intoxication: An updated review. J. Neurol Sci..

[18] Ma Y.F., Yu G., Zhang J.B., Yu X., Sun R. (2015). Sensitive detection of carbon monoxide based on a QEPAS sensor with a 2.3 μm fiber-coupled antimonide diode laser. J. Opt. UK.

[19] Kosterev A.A., Tittel F.K. (2004). Ammonia detection using quartz-enhanced photoacoustic spectroscopy with a near-IR telecommunication diode laser. Appl. Opt..

[20] Zheng H.D., Dong L., Liu X.L., Liu Y.Y., Wu H.P., Ma W.G., Zhang L., Yin W.B., Jia S.T. (2015). Near-IR telecommunication diode laser based double-pass QEPAS sensor for atmospheric CO_2_ detection. Laser Phys..

[21] Spagnolo V., Patimisco P., Borri S., Scamarcio G., Bernacki B.E., Kriesel J. (2013). Mid-infrared fiber-coupled QCL-QEPAS sensor. Appl. Phys. B.

[22] Spagnolo V., Patimisco P., Pennetta R., Sampaolo A., Scamarcio G., Vitiello M.S., Tittel F.K. (2015). THz Quartz-enhanced photoacoustic sensor for H_2_S trace gas detection. Opt. Express.

[23] Borri S., Patimisco P., Sampaolo A., Beere H.E., Ritchie D.A., Vitiello M.S., Scamarcio G., Spagnolo V. (2013). Terahertz quartz enhanced photo-acoustic sensor. Appl. Phys. Lett..

[24] Wu H.P., Sampaolo A., Dong L., Patimisco P., Liu X.L., Zheng H.D., Yin X.K., Ma W.G., Zhang L., Yin W.B. (2015). Quartz enhanced photoacoustic H_2_S gas sensor based on a fiber-amplifier source and a custom tuning fork with large prong spacing. Appl. Phys. Lett..

[25] Wu H.P., Dong L., Zheng H.D., Liu X.L., Ying X.K., Ma W.G., Zhang L., Yin W.B., Jia S.T., Tittel F.K. (2015). Enhanced near-infrared QEPAS sensor for sub-ppm level H_2_S detection by means of a fiber amplified 1582 nm DFB laser. Sens. Actuators B Chem..

[26] Wu H.P., Dong L., Liu X.L., Zheng H.D., Yin X.K., Ma W.G., Zhang L., Yin W.B., Jia S.T. (2015). Fiber-Amplifier-Enhanced QEPAS Sensor for Simultaneous Trace Gas Detection of NH_3_ and H_2_S. Sensors.

[27] Spagnolo V., Patimisco P., Borri S., Scamarcio G., Bernacki B.E., Kriesel J. (2012). Part-per-trillion level SF_6_ detection using a quartz enhanced photoacoustic spectroscopy-based sensor with single-mode fiber-coupled quantum cascade laser excitation. Opt. Lett..

[28] The HITRAN Database. http://www.hitran.com.

[29] Dong L., Kosterev A.A., Thomazy D., Tittel F.K. (2010). QEPAS spectrophones: Design, optimization, and performance. Appl. Phys. B.

[30] Dong L., Lewicki R., Liu K., Buerki P.R., Weida M.J., Tittel F.K. (2012). Ultra-sensitive carbon monoxide detection by using EC-QCL based quartz-enhanced photoacoustic spectroscopy. Appl. Phys. B.

[31] Kosterev A.A., Tittel F.K., Serebryakov D.V., Malinovsky A.L., Morozov I.V. (2005). Applications of quartz tuning forks in spectroscopic gas sensing. Rev. Sci. Instrum..

[32] Kosterev A.A., Bakhirkin Y.A., Tittel F.K., McWhorter S., Ashcraft B. (2008). QEPAS methane sensor performance for humidified gases. Appl. Phys. B.

[33] Pao Y.H. (1977). Optoacoustic Spectroscopy and Detection.

